# Artificial Intelligence: A New Frontier in Rare Disease Early Diagnosis

**DOI:** 10.7759/cureus.79487

**Published:** 2025-02-22

**Authors:** Syed Muhammad Hayyan Nishat, Ammar Shahid Tanweer, Bashayer Alshamsi, Majd H Shaheen, Ariba Shahid Tanveer, Aroob Nishat, Yaman Alharbat, Ahmad Alaboud, Mahra Almazrouei, Raghad A Ali-Mohamed

**Affiliations:** 1 Internal Medicine, RAK Medical & Health Sciences University, Ras Al Khaimah, ARE; 2 Medicine, RAK Medical & Health Sciences University, Ras Al Khaimah, ARE; 3 Pediatrics, Fujairah Hospital, Fujairah, ARE

**Keywords:** artificial intelligence, early identification and diagnosis, genetic analysis, machine learning, rare diseases

## Abstract

Rare diseases present significant challenges, including delays in diagnosis, inadequate treatment responses, and difficulties in monitoring. These challenges arise from the complexity of symptoms, limited medical expertise, and insufficient diagnostic tools. Artificial Intelligence (AI) has gained attention for its potential to improve healthcare, particularly in diagnosing complex conditions. By analyzing large datasets, recognizing patterns, and integrating clinical information, AI can refine diagnostic accuracy, enhance treatment strategies, and improve patient outcomes.

This literature review examines AI applications in three key areas of rare disease diagnosis: genetic analysis, imaging-based phenotyping, and natural language processing (NLP) for clinical data extraction. AI-driven advancements in these domains have been explored to improve disease detection and management. However, several challenges persist, including limited data availability, algorithm transparency, privacy considerations, and ethical concerns. Efforts such as data augmentation and transfer learning are being explored to address these issues and expand AI’s role in clinical practice.

By refining diagnostic processes and optimizing treatment strategies, AI has the potential to improve the management of rare diseases. This review critically examines AI's role in rare disease diagnosis, with a particular emphasis on its applications in genetic analysis, imaging-based phenotyping, and NLP, while also addressing key challenges and future directions for clinical integration.

## Introduction and background

More than 350 million people worldwide are afflicted by more than 7,000 rare diseases (RDs), each of which affects a comparatively small number of patients. However, obtaining a timely and accurate diagnosis remains a significant challenge. Patients with rare disorders typically search for a proper diagnosis for six years after their symptoms first appear [1]. According to a 2013 survey, patients with uncommon diseases often must wait an average of more than five years, seeing eight different doctors and going through two to three misdiagnoses before a proper diagnosis is made [2]. Moreover, RDs are difficult for physicians to diagnose quickly because of their complexity, unpredictability, lack of specific symptoms, and general lack of knowledge about such conditions [3].

These challenges arise due to the low prevalence of specific rare diseases, a scarcity of experts, limited accessibility to specialists, and inadequate infrastructure and funding for RD research. The use of computer-based support to enhance clinical workflows is an emerging technology, especially in the fields of AI and machine learning (ML), to overcome these challenges [1,2]. Furthermore, ML methods have been applied to phenotyping and drug discovery, as well as a number of healthcare domains, such as disease diagnosis, prediction of disease onset, mortality, and length of hospital stay. Consequently, using deep learning (DL) to solve the complexity related to rare diseases has garnered a lot of interest [1].

AI refers to the capability of machines to perform tasks that typically require human intelligence, including learning, problem-solving, and decision-making. Numerous AI algorithms provide noteworthy advantages in supporting the diagnosis of RDs and non-RDs. A branch of AI called ML allows machines to learn from their experiences and become more efficient without the need for explicit programming. Through three main types of algorithms, ML plays a critical role in diagnosis: (a) unsupervised, which recognizes patterns; (b) supervised, which classifies or predicts based on prior examples; and (c) reinforcement learning, which uses reward and punishment processes to generate strategies for overcoming particular obstacles. A subset of ML techniques called DL is focused on picture recognition. By breaking down complex mappings into groups of simpler ones, DL allows for more effective analysis [4]. Finally, a large amount of the human variability and bias inherent in translating data from medical records can be eliminated by using natural language processing (NLP), a branch of AI that focuses on understanding human language [5].

Focus of this review & research question

While AI has been broadly applied to various areas of healthcare, this review specifically focuses on three critical applications of AI in rare disease diagnosis: (1) genetic analysis, (2) imaging-based phenotyping, and (3) natural language processing (NLP) for clinical data extraction. These areas were chosen because they represent the most promising and actively researched AI-driven approaches for addressing the challenges of rare disease diagnosis.

Given AI's growing role in rare disease diagnosis, this review seeks to answer the following question: How has AI contributed to early and accurate rare disease diagnosis, and what challenges must be overcome for its broader clinical adoption? To address this question, the review examines AI applications in these three domains:

1. Genetic Analysis: Many rare diseases have a genetic basis, and AI-driven approaches have been developed to enhance the interpretation of genomic data, identify pathogenic variants, and improve diagnostic accuracy.

2. Imaging-Based Phenotyping: AI, particularly deep learning, has shown significant promise in identifying disease-associated patterns in medical imaging, enabling earlier and more accurate detection of rare diseases.

3. NLP: AI-powered NLP techniques can extract meaningful insights from unstructured clinical data, including physician notes and electronic health records, to support the identification of rare diseases that might otherwise be missed.

By systematically analyzing AI’s contributions, current limitations, and future research directions in these three domains, this review provides a structured evaluation of AI’s role in rare disease diagnosis and explores the necessary steps for its broader clinical adoption.

**Figure 1:**
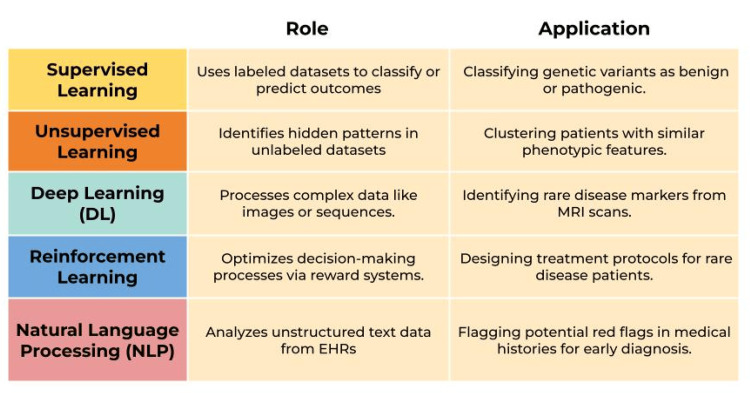
AI Techniques in Rare Disease Diagnosis AI: Artificial Intelligence; EHR: Electronic Health Records Image Credits: Syed Muhammad Hayyan Nishat

Figure 1 illustrates the various applications of AI and ML in problem-solving and decision-making processes.

To diagnose RDs, first-generation AI focuses on making clinical choices by evaluating large datasets. However, one of the biggest obstacles facing RDs is the lack of initial data inputs. On the other hand, second-generation AI looks for clinical hints that are frequently missed in the initial phases of RD detection. This approach efficiently supports early diagnosis, enabling intervention and prevention for a range of RDs. For example, ML has been used recently to identify individuals with systemic sclerosis who are at a high risk of serious consequences and to identify organ involvement early [4].

AI and ML are currently being used in healthcare settings to improve medical image processing, forecast and prevent illnesses, and optimize hospital operations. Healthcare professionals can diagnose and treat patients more accurately and efficiently by utilizing these technologies. Moreover, doctors may be able to diagnose, treat, and manage patients more quickly with the help of AI and ML, which would eventually improve patient outcomes [4].

This article explores the innovative potential of AI in aiding healthcare professionals to identify patients promptly and accurately with rare diseases, consequently, lessening the time required for diagnosis and thus enhancing their chances of receiving timely medical attention.

## Review

Methodology

This review was conducted by manually collecting relevant literature from multiple databases, including PubMed, ScienceDirect, UpToDate, and Google Scholar, covering studies published between January 2013 and January 2025. The following keywords were used to identify relevant articles: “Artificial Intelligence”, “AI”, “Screening”, “Rare Diseases”, “ultra-rare disease”, “machine learning”, “deep learning”, and “Orphan diseases”.

**Figure 2:**
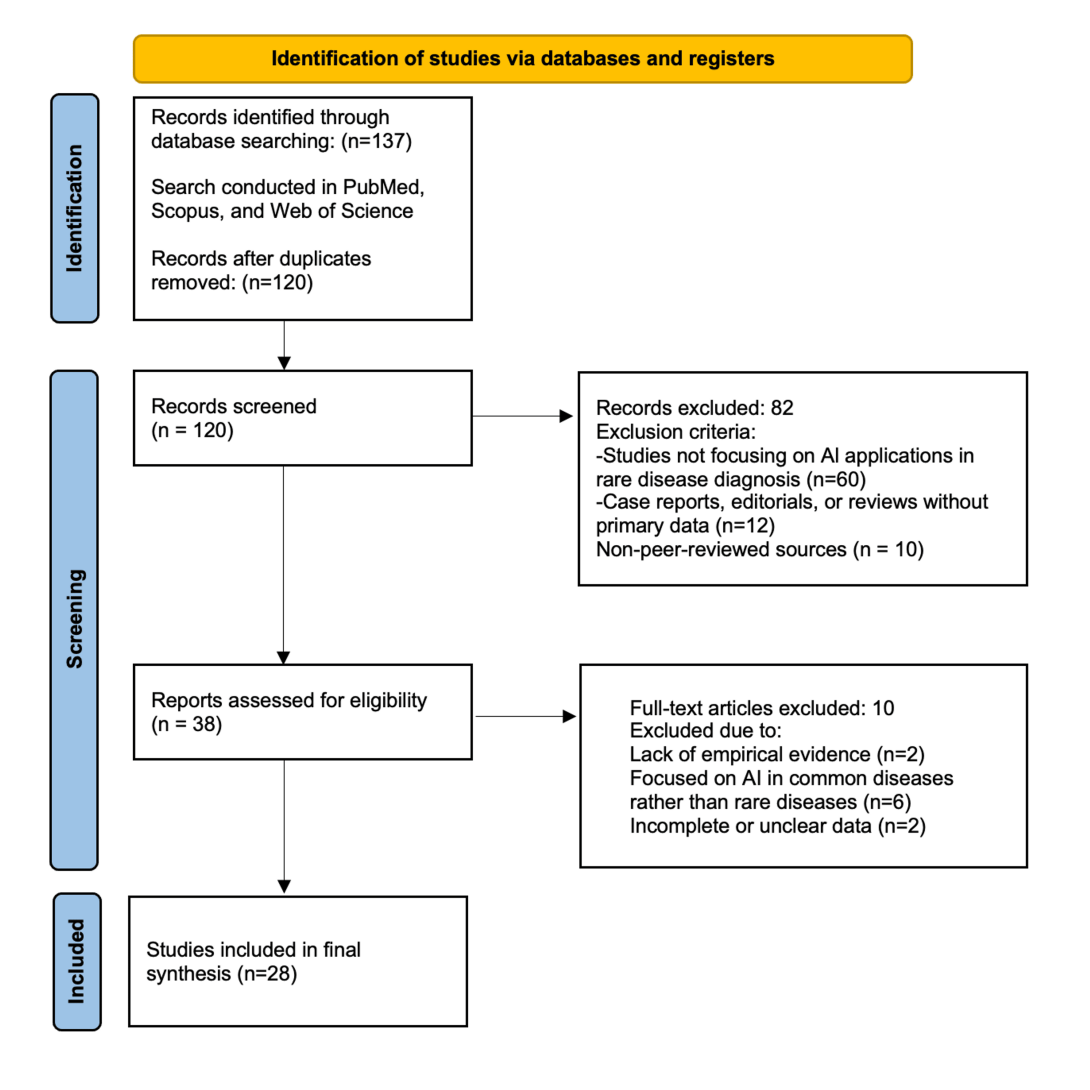
PRISMA flowchart of selected articles. PRISMA: Preferred Reporting Items for Systematic Reviews and Meta-Analyses.

A total of 28 articles met the inclusion criteria for this review. Studies were selected based on their relevance to AI applications in rare disease diagnosis, with a focus on genetic analysis, imaging-based phenotyping, and NLP. Articles were included if they were peer-reviewed, published in English, and provided empirical evidence, systematic reviews, or advancements in AI methodologies related to rare disease diagnosis. Studies that focused solely on AI applications in common diseases, did not discuss genetic analysis, imaging, or NLP, or were preprints, opinion pieces, or non-peer-reviewed sources were excluded. The study selection process is illustrated in the PRISMA flowchart (Figure 2).

The selected studies were analyzed to identify common trends, advancements, limitations, and future directions in AI-driven rare disease diagnosis. This qualitative synthesis provides an overview of AI’s impact on rare disease diagnostics across genetic, imaging, and NLP-based approaches.

Risk of Bias Assessment

A general assessment of potential biases in the included studies was conducted based on key methodological factors such as selection criteria, study design, data sources, and funding disclosures. While no formal risk of bias tool was applied, considerations were made regarding potential limitations that could influence the findings.

Overall, some studies exhibited limitations related to sample size, potential conflicts of interest, and selective reporting. However, efforts were made to include peer-reviewed studies and reputable sources to enhance the credibility of this review. Future research should aim to adopt more standardized methodologies to minimize bias and improve reproducibility.

AI in the genetic diagnosis of rare diseases

AI has emerged as a powerful tool in rare disease diagnosis, integrating diverse data sources to enhance accuracy and early detection​ [6]. It is currently regularly employed, and a part of diagnostic workups in highly specialized centers for the diagnosis and management of diseases in oncology, rheumatology, and genetics [7]. Over the past decade, there has been increasing interest in the application of these technologies and methods to aid in the diagnosis of rare diseases.

**Figure 3:**
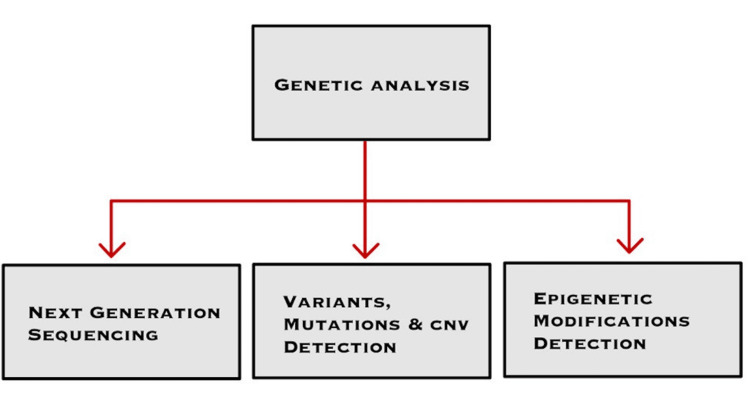
Genetic Analysis Flowchart CNV: Copy Number Variations Image Credits: Ammar Shahid Tanweer

Since 80% of rare diseases in literature have an underlying genetic cause, the application of AI has proved to be quite useful [4]. Next Generation Sequencing (NGS) is the process of sorting through millions of DNA sequences, which when combined with AI can help in the detection of genetic anomalies [7]. AI algorithms play an important role in enhancing and automating various aspects of NGS data analysis, leading to increased precision and efficiency. One of the most important applications of AI in NGS data analysis is to align the sequences to a reference genome which is crucial for the detection of gene variations and mutations. AI excels in this task and facilitates the development of novel tools for NGS data interpretation [8]. Figure 3 provides a visual representation of the key steps involved in comprehensive genetic analysis.

Many different tools have been developed using NGS to aid in the diagnosis of Mendelian diseases. PhenIX is one such tool which integrates genetic analysis with phenotypic appearance. It reads the patient's genotype, finds any genetic variants and then prioritizes these based on the phenotypic appearance of the patient. Another tool, Xrare does the same [4]. In addition to this, these tools can predict the pathogenicity of genes, discover molecular markers, and create predictive models based on gene expression data. AI can quickly scan and sort through large genetic datasets, which is not possible by humans [4].

A study assessed Fabric GEM (Fabric Genomics, Oakland, CA, USA), a new AI-based tool for hastening genome interpretation (whole-genome sequencing (WGS) and whole-exome sequencing (WES)), on a retrospective cohort of 119 patients diagnosed with rare genetic diseases in addition to a separate cohort of 60 cases from different academic centers. GEM identified over 90% of the causative genes as the top or second candidate and identified the causative structural variant (SV) as the top candidate in 17 of 20 patients with diagnostic SVs [9].

AI, especially ML, has also been applied to the field of epigenetics. Epigenetic modifications have been identified in many rare diseases including Rett syndrome, hereditary sensory autonomic neuropathy type 1E, Cornelia de Lange syndrome, Angelman syndrome, Prader-Willi syndrome, and Beckwith-Wiedemann syndrome [10, 11].

Natural language processing in rare disease diagnosis

The increasing adoption of electronic health records (EHRs) globally is advancing data mining in healthcare. Data from EHRs can be structured (as in constrained choices via drop-down menus, check boxes, and pre-filled templates) and unstructured (as in free text narratives/notes used by healthcare professionals) [12].

This is a promising field where data from these sources can be used to develop models to support early diagnosis. NLP is a subset of AI which is concerned with understanding, analyzing, and interpreting the human language [5]. NLP technology can be used along with Unified Medical Language System (UMLS) to convert health providers’ notes and narratives into structured, standardized formats, which can then further be interpreted for establishing natural history and assisting in early diagnosis of a variety of diseases, including rare diseases [12]. This approach is based on the premise that these data capture unique insights from experienced physicians, which might otherwise be overlooked in formal measurements or purely biological assessments [13].

A study conducted used NLP and UMLS to study a cohort of Dravet syndrome (DS) which presents with the onset of epileptic encephalopathy in the first year of life, characterized by febrile seizures and convulsive status epilepticus, and a cohort of febrile seizures not linked to an underlying disorder. The results showed the presence of certain keywords and concepts specific to DS diagnosis in the medical records which were automatically detected by the software using NLP. This approach represents a new methodology that can help flag such symptoms and decrease the time to diagnose DS and could also be extrapolated to other rare diseases [12].

Another study validated a novel, state-of-the-art NLP algorithm for the detection of red flag symptoms of multisystemic hereditary transthyretin amyloidosis (ATTRv) polyneuropathy from EHRs, in a retrospective sample of 1015 patients. The accuracy of the algorithm was compared with a manual standard on a random sample of 300 patients. There was high accuracy in the detection of red flag symptoms, which showed F1 scores between 0.88 and 0.98. There was a relative increase of 48.6% in genetic testing, which has implications for earlier detection of patients with a rare disease [14].

AI has been used for retrospective screening of over 300,000 unstructured EHRs for flagging patients for Pompe’s disease and Mucopolysaccharidosis type II (MPS-II). The algorithms had a specificity of 18.27% for Pompe’s disease, which is crucial as it is a progressive but treatable debilitating neuromuscular disease [15]. The algorithms had a very high accuracy (99%) and sensitivity (84%) for MPS-II [10].

AI-assisted phenotype imaging analysis in the diagnosis of rare diseases

Many rare diseases exhibit distinctive dysmorphic features [10]. DL is a subset of ML which is involved in image processing. DL algorithms can break down complex imaging mappings into simpler mappings which allows for highly efficient analysis. The algorithm then uses these simpler mappings to read the image and derive important information from it. DL provides superior recognition and can process large amounts of data in a very short time, which can aid in a swift and accurate diagnosis. AI has been shown to have a higher flexibility and scalability as compared to traditional biometrics, which has contributed to early detection, superior efficiency and understanding of complex relationships [4].

DeepGestalt (FDNA, Atlanta, GA, USA) is one such deep-learning facial analysis framework used for the classification of genetic diseases. DeepGestalt has been applied in multiple studies for rare disease diagnosis, demonstrating moderate to high accuracy. These diseases include Cornelia de Lange syndrome, Emanuel syndrome, and Pallister-Killian syndrome. It can differentiate between 200 RDs and is able to achieve 91% accuracy in getting the correct diagnosis in the top 10 results [10,16]. In one study, DeepGestalt was applied for the analysis of facial phenotypes of 25 individuals with KBG syndrome. KBG syndrome was identified as the first (most probable) diagnosis in 28% of patients, second in 40%, and third or fourth for 12%. Overall, KBG syndrome was identified as a potential diagnosis as the top five differentials in 80% of individuals [17].

**Figure 4:**
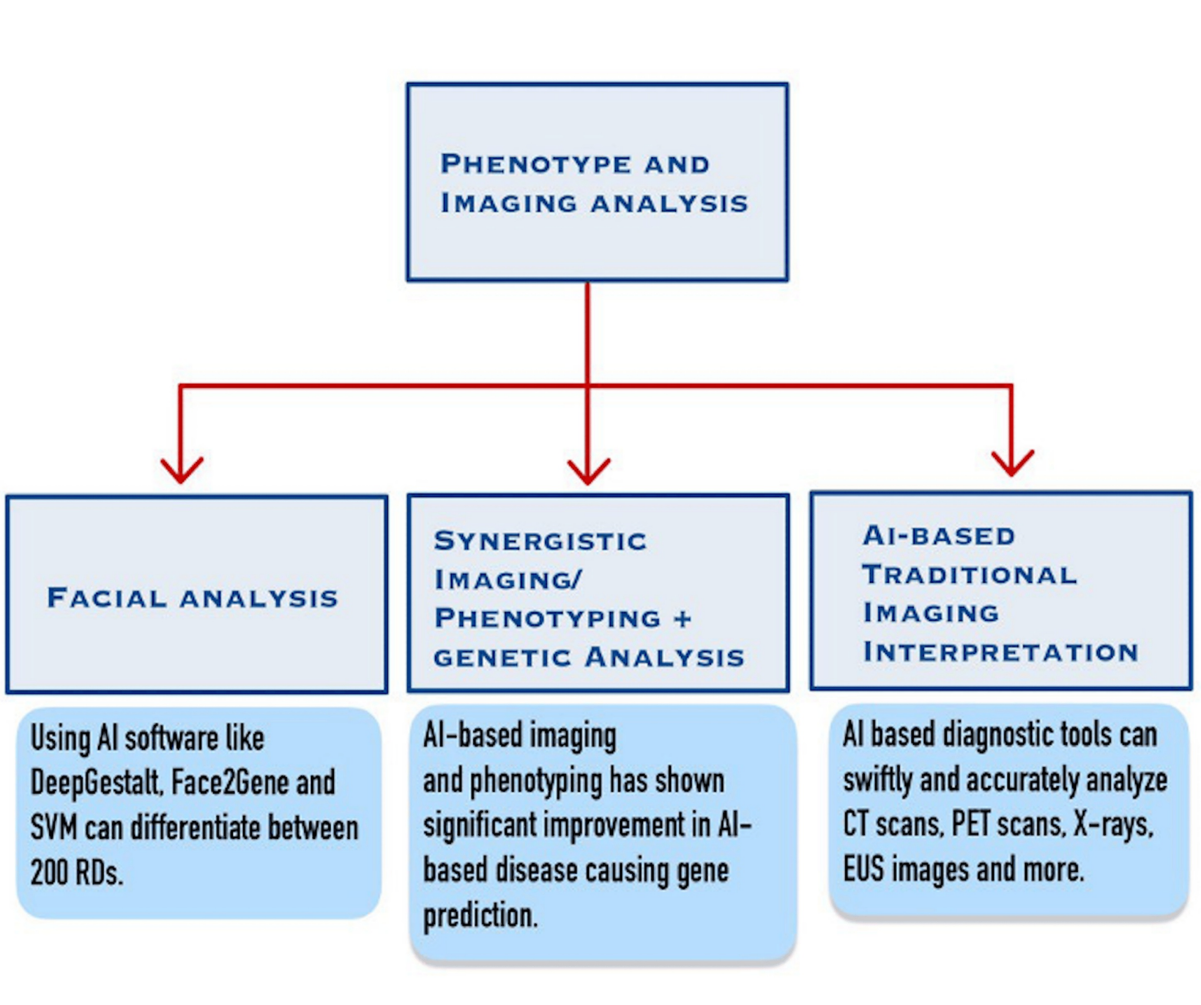
Applications of Phenotype and Imaging Analysis SVM: Support Vector Machines; PET: Positron Emission Tomography; EUS: Endoscopic Ultrasound Image Credits: Ammar Shahid Tanweer

Face2Gene (FDNA, Atlanta, GA, USA) is another software that uses DL to analyze facial photographs and help in the diagnosis of rare diseases [18]. Other algorithms like Support Vector Machines (SVM) have been used in the diagnosis of rare diseases like acromegaly, Pick disease and amyotrophic lateral sclerosis (ALS) [10]. Figure 4 illustrates three applications of phenotype and imaging analysis.

Imaging can be combined with other diagnostic methods synergistically, improving the diagnostic potential of AI. For example, imaging analysis using AI enhances the results of AI NGS-based genetic analysis and provides a higher level of accuracy in the prediction of pathogenic genes. This approach is called PEDIA (Prioritization of Exome Data by Image Analysis) and was tested in a large cohort of patients with monogenic rare diseases, showing significantly improved disease-causing gene prediction [10]. Similarly, a combination of AI image analysis and brain function was used for determining if a patient with Huntington's disease (HD) will receive a clinical diagnosis within the next five years, and for assessment of oculomotor function preceding HD [4]. Another framework, Phen-Gen, shows very promising results by combining patients’ sequencing data and phenotypes for the recognition of coding and non-coding variants in rare diseases. It showed a significant 52% increased accuracy in variant prediction as compared to genotype-only analyses [10].

ML and DL have been instrumental in the development of diagnostic tools that can swiftly analyze CT scans, X-ray images, and images from other modalities to identify patterns characteristic of diseases [18]. AI-based positron emission tomography (PET) has huge potential in the diagnosis of rare diseases as well. It has shown to have a significant role in the diagnosis and subsequent management of multiple rare diseases including multiple endocrine neoplasias, pheochromocytoma and paraganglioma (PPGL), Von Hippel-Lindau disease, Erdheim-Chester disease, familial carcinoid syndrome and Duchenne muscular dystrophy (DMD) [16].

AI deep learning algorithms, convolutional neural networks (CNN) and recurrent neural networks (RNN), have achieved very good results in image classification and radiomics for cholangiocarcinoma [19]. A retrospective study tested AI analysis of endoscopic ultrasound (EUS) images of intraductal papillary mucinous neoplasms (IPMNs) of the pancreas to predict malignancy, and results revealed a very high accuracy (94%) as compared to human diagnosis (54%) [20].

ML has been shown to be highly effective in the diagnosis of systemic sclerosis. ML outperforms traditional standard diagnostic approaches like high-resolution computed tomography (HRCT) and pulmonary function tests (PFTs) for detecting pulmonary involvement. ML algorithms could identify pulmonary involvement before deterioration occurs, which can improve survival rates and lower medical expenses [4].

Functional magnetic resonance imaging (fMRI) and diffusion tensor imaging reveal similarities in brain function that suggest overlap across several neurodevelopmental disorders (NDD) at the intermediate phenotypic level. ML-based supervised and unsupervised analysis of biological datasets may produce predictive models for differentiating between these NDDs and aid in both the diagnosis and treatment of these disorders which include some RDs [13].

AI models utilizing a variety of different techniques to identify and classify genetic mutations in RDs have been highlighted in Tables 1, 2.

**Table 1:** Overview of AI/ML-Based Genetic Diagnostic Tools and Their Applications in Rare Disease Research VEST: Variant Effect Scoring Tool; SilVA: Silent Variant Analyzer Tool; SNAP: Screening for Non-Acceptable Polymorphisms Tool; PennCNV: A model for detecting copy number variations in whole-genome single nucleotide polymorphisms data; SpliceAI: Deep neural network tool for predicting splicing from pre-mRNA sequences; VarCoPP: Variant Combinations Pathogenicity Predictor Tool; CNVdigest: A web tool that uses text mining to link copy number variations with genetic diseases; eDIVA: Exome Disease Variant Analysis Tool; CF: Cystic Fibrosis; PKU: Phenylketonuria; HOCM: Hypertrophic Obstructive Cardiomyopathy; CVID: Common Variable Immunodeficiency; ML: Machine Learning; AI: Artificial Intelligence; CNV: Copy Number Variation; SNPs: Single Nucleotide Polymorphisms; RDs: Rare Diseases

Author name	Year of publication	Software/ Algorithm/ Platform	Rare Disease	Genetic Diagnostic Method	Result
Carter et al. [21]	2013	VEST	Freeman Sheldon Syndrome	Single Nucleotide Variants	AI scoring tool (VEST) placed the causal gene among the top 2.
Buske et al. [22]	2013	SilVA	Meckel Syndrome	Single Nucleotide Variants	SilVA can accurately predict the harmfulness of silent variants in Meckel syndrome and other rare diseases.
Browne and Timson [23]	2015	SNAP	Mevalonate Kinase Deficiency	Single Nucleotide Variants	AI-based models analyzed and sorted gene variants and the effect they produced into three groups - mild, intermediate and severe.
Orange et al. [24]	2011	PennCNV	Common Variable Immunodeficiency	Single Nucleotide Variants	363 CVID patients were genotyped, showing 610,000 SNPs, 1000 of which were strongly predictive of CVID.
Jaganathan et al. [25]	2019	SpliceAI	HOCM	Slicing and Multigenic mutations	SpliceAI, a NN, was used to assess the impact of a pathogenic cryptic splice mutation in HOCM. The analysis of such mutations opens a new avenue for the diagnosis of RDs.
Papadimitriou et al. [26]	2019	VarCoPP	Oligogenic Diseases	Slicing and Multigenic mutations	VarCoPP uses ML and was trained using known rare oligogenic diseases and had high confidence labels of 95% and 99%.
Yang et al. [27]	2018	CNVdigest	DiGeorge Syndrome	Copy Number Variation	DNorm, an AI ML-powered tool, to generate CNVdigest, which contains 1582 CNVs and 2425 diseases. CNVdigest identifies CNV-disease associations and has shown results with DiGeorge syndrome.
Bosio et al. [28]	2019	eDiVA	Several diseases including CF, PKU	Genotype-Phenotype integration	An AI tool eDiVA was applied to rare diseases like CF, PKU among others. It was able to identify known causal variants with high precision and recall.

**Table 2:** Comparative Overview of AI Performance in Rare Disease Diagnosis ROC AUC (Receiver Operating Characteristic - Area Under Curve): Measures how well the AI model distinguishes between disease-causing and benign variants. AUC = 1.0 is perfect, while 0.5 is random chance. Accuracy (%): Proportion of correctly classified cases, including disease-causing and benign variants. F1 Score: A balance between precision (correct positive predictions) and recall (ability to detect all actual cases). Higher values indicate better model performance, especially for rare diseases. Sensitivity (%): The ability to correctly detect true positive cases (e.g., AI-based PET imaging detects >95% of rare metabolic cancers). Top-N Diagnosis Accuracy: The percentage of times the correct diagnosis appears in the top N results (e.g., DeepGestalt correctly includes the diagnosis 91% of the time in its top 10 suggestions).

AI Technique	Rare Disease(s) Applied	Performance Metrics	Efficiency	Clinical Utility
Fabric GEM (Genomic AI Tool)	Rare genetic disorders	>90% accuracy (gene identification)	Rapid genome interpretation	Used in genetic centers for WGS/WES analysis [9]
Phen-Gen (AI-driven Genotype-Phenotype Integration)	Monogenic Rare Diseases	52% increased accuracy in gene prediction	Integrates AI-driven imaging with genetic data	Enhances diagnostic accuracy when combined with imaging [10]
NLP for EHRs (UMLS-based AI models)	Dravet Syndrome, ATTRv polyneuropathy	F1 score: 0.88 - 0.98	Automated medical text mining	Increases genetic testing rates [14]
DeepGestalt (DL Facial Analysis)	Cornelia de Lange, KBG syndrome, Monogenic RDs	91% top-10 diagnosis accuracy	Automated facial phenotype recognition	Used in dysmorphology clinics [10,16]
Medical Imaging AI (Radiomics, CNN/RNN models, PET AI)	Cholangiocarcinoma, Huntington’s Disease, Pheochromocytoma	>95% sensitivity (PET AI), 94% accuracy (IPMN AI)	Fast, automated image classification	Used in oncology and metabolic disorders [16]
VEST (Variant Effect Scoring Tool)	Mendelian disorders (e.g., Miller Syndrome, Freeman-Sheldon Syndrome)	ROC AUC: 0.91	Prioritizes disease-causing genetic variants	Used in genetic variant analysis [21]
SilVA (Silent Variant Analyzer)	Meckel Syndrome, silent mutation analysis	46% success rate in ranking pathogenic variants in top 5	Prioritizes synonymous variants	Identifies silent variants affecting splicing [22]
SpliceAI (Deep Neural Network for Splicing Mutations)	Hypertrophic Cardiomyopathy	>95% precision in pathogenic splice mutation prediction	High accuracy in detecting cryptic splice sites	Helps in early molecular diagnosis [25]
VarCoPP (Variant Combination Pathogenicity Predictor)	Oligogenic Diseases	95-99% confidence in oligogenic disease markers	High reliability for pathogenic variant detection	Applied in rare disease classification [26]
CNVdigest (CNV Database & AI Model)	DiGeorge Syndrome, Copy Number Variants (CNVs)	Extensive CNV-disease relationship mapping	Systematic text mining	Provides a knowledge base for CNV-disease studies [27]
eDiVA (Enhanced Diagnostic Variant Analysis)	Cystic Fibrosis, Phenylketonuria	Accuracy: 89%	Automated variant prioritization	Used in clinical genetic diagnostics [28]

Challenges in implementing AI

Scarcity of Data

AI models have inherent limitations, particularly those requiring large, high-quality datasets for optimal performance. This problem is particularly apparent in the case of RDs, as there is frequently insufficient data available for model training [4].

The scarcity of data poses a significant obstacle in deep learning research for rare diseases, as highlighted in analyses of the challenges associated with employing this technology in this domain [1]. With their abundance of omics data and small patient population, RDs pose a distinct data issue. Because orphan patients are still uncommon, ML analyses are still greatly impacted by this, even with registries. In order to support modeling efforts, open data can therefore be quite important. In the future, utilizing open data and patient registries together will be essential to extracting as much information as possible for possible insights [6]. Additionally, the complexity of treating numerous RDs is further exacerbated by the variety of clinical presentations, therapeutic modalities used by various healthcare professionals, and variations in biological traits [4]. It's imperative to conduct more research on RDs to gather additional data.

Several methods based on deep learning have been used in papers to solve this problem. A number of studies have concentrated on creating novel deep-learning architectures that are suited to deal with the shortage of data. Data augmentation is another frequently employed approach to address the issue of limited data, and it will be covered in more detail in overcoming limitations [1].

Lack of Interpretability

A key challenge is the lack of interpretability in AI models, making it difficult for researchers and physicians to understand algorithmic outputs. Explainability is still an issue of contention when it comes to using AI in healthcare. Even though AI-powered systems have outperformed humans in several analytical tasks, explainability is still lacking, which is a source of criticism. In this sense, explainable AI techniques mark the next wave of machine learning applications in the medical field, with the goal of making sure that patients and doctors alike are aware of how artificial intelligence makes decisions [6].

One significant challenge faced by supervised AI models, particularly in natural language processing, is their difficulty in distinguishing between sentences with opposite meanings that share similar wording [29]. This phenomenon, known as semantic ambiguity, poses a critical issue in medical AI applications, where minor variations in phrasing can lead to vastly different clinical interpretations [29]. For example, a model might struggle to differentiate between ‘The patient is not experiencing symptoms of infection’ and ‘The patient is now experiencing symptoms of infection,’ despite their structural similarity [29]. This limitation arises because many supervised models rely heavily on word frequency and syntactic patterns rather than deep contextual understanding [29]. Addressing this challenge requires context-aware AI approaches, such as transformer-based models, which utilize attention mechanisms to capture nuanced semantic differences [29]. Despite these advancements, ambiguity remains a persistent issue in NLP applications for healthcare and rare disease diagnosis, as highlighted in recent literature.

Privacy Issues

The sharing of incidental discoveries made by AI systems raises consent issues, especially when it comes to rare diseases. Strict regulations are necessary to prevent the misuse of AI-generated data, especially in vulnerable populations. This covers steps to guarantee the moral application of AI technology in healthcare contexts as well as patient privacy protection [18].

Specific Limitations

The application of AI in healthcare poses several ethical, legal, technological, and societal problems. Data ethics, privacy, and potential biases in AI systems are ethical issues that affect clinical decision-making and the management of patient health data. The absence of international AI laws and duties in AI healthcare applications leads to legal complications. To stop attacks on AI systems and data breaches, security precautions are crucial. The degree of societal acceptability varies; patients frequently favor medical professionals' diagnosis over those made by artificial intelligence, and healthcare workers in developing nations express concerns about their job security [18].

Scientific problems include algorithmic fairness, which involves bias, dataset shifts which include variations in the properties or distribution of data over time, and adaptation to changing healthcare practices. Resolving biases in AI systems is essential for providing healthcare in a fair manner [18].

Overcoming the limitations

Recent advancements have introduced solutions to data limitations, such as data augmentation. The model can be trained, and patterns can be found from a smaller dataset by using this technique, which can either modify the original data or create synthetic data. This is very useful when working with restricted data. Patterns can even be identified from complex and unlabeled data by using an unsupervised learning technique [1,4].

Fine-tuning, or transfer learning, involves training AI models on a large dataset (e.g., common diseases) before refining them using smaller datasets (e.g., rare diseases). For instance, the PLIER framework developed by researchers utilizes an unsupervised transfer learning framework and can be adapted to smaller datasets such as RDs and precision medicine [4].

Randomized controlled trials (RCTs) that undergo peer review are crucial for promoting trust and AI adoption in medicine. It is necessary to evaluate biases and utility in clinical practice by transparent reporting of AI research and adherence to known best practices, such as the Transparent Reporting of a Multivariable Prediction Model for Individual Prognosis or Diagnosis (TRIPOD) [18].

## Conclusions

To summarize, the integration of AI and ML in the domain of RDs provides an excellent opportunity for substantial progressions in patient care, diagnosis, and treatment. With their wide range of appearances and rarity, RDs have traditionally presented difficult challenges. However, with the integration of AI and ML with a wealth of biomedical knowledge and data, RDs are about to undergo a radical change. AI is particularly useful in the diagnosis field since it can quickly identify rare diseases by evaluating large datasets from multiple sources. These technologies efficiently solve issues resulting from a lack of knowledge and awareness by classifying patients into illness groups, analyzing symptoms, and even using facial scans for diagnosis. AI additionally makes precision medicine achievable by using patient registry data to personalize care and follow-up according to individual genetic profiles. This improves diagnosis, treatment outcomes, and prevention while offering valuable knowledge about the well-being of the patient.

However, there are challenges and constraints associated with the application of AI in healthcare that are related to legal, ethical, and technological issues like security, privacy, bias, and data ethics. It is critical to overcome these obstacles while maintaining open lines of communication and attending to concerns about data safety. Despite these obstacles, the future of AI and ML in RDs holds great promise. As AI applications continue to advance and health databases expand, personalized, efficient, and patient-centered care is becoming increasingly achievable. AI complements human intelligence to significantly enhance the lives of individuals with RDs, ultimately reducing the burden on both patients and healthcare systems.
